# On the accuracy of cell-attached current-clamp recordings from cortical neurons

**DOI:** 10.3389/fnmol.2022.979479

**Published:** 2022-08-11

**Authors:** Alina Vazetdinova, Fliza Valiullina-Rakhmatullina, Andrei Rozov, Alexander Evstifeev, Roustem Khazipov, Azat Nasretdinov

**Affiliations:** ^1^Laboratory of Neurobiology, Kazan Federal University, Kazan, Russia; ^2^Institut für Physiologie und Pathophysiologie, Heidelberg, Germany; ^3^Federal Center of Brain Research and Neurotechnologies, Moscow, Russia; ^4^INMED - INSERM, Aix-Marseille University, Marseille, France

**Keywords:** patch-clamp technique, cell-attached, neurons, cortex, hippocampus, depolarizing action of GABA, giant depolarizing potentials (GDPs), sharp wave ripple

## Abstract

Cell-attached current-clamp (CA/CC) recordings have been proposed to measure resting membrane potential and synaptic/agonist responses in neurons without disrupting the cell membrane, thus avoiding the intracellular dialysis that occurs in conventional whole-cell recordings (WC). However, the accuracy of CA/CC recordings in neurons has not been directly assessed. Here, we used concomitant CA and WC current clamp recordings from cortical neurons in brain slices. Resting membrane potential values and slow voltage shifts showed variability and were typically attenuated during CA/CC recordings by ~10–20% relative to WC values. Fast signals were slowed down and their amplitude was greatly reduced: synaptic potentials by nearly 2-fold, and action potentials by nearly 10-fold in CA/CC mode compared to WC. The polarity of GABAergic postsynaptic responses in CA/CC mode matched the responses in WC, and depolarising GABAergic potentials were predominantly observed during CA/CC recordings of intact neonatal CA3 hippocampal pyramidal neurons. Similarly, CA/CC recordings reliably detected neuronal depolarization and excitation during network-induced giant depolarizing potentials in the neonatal CA3 hippocampus, and revealed variable changes, from depolarization to hyperpolarization, in CA1 pyramidal cells during sharp wave ripples in the adult hippocampus. Thus, CA/CC recordings are suitable for assessing membrane potential but signal distortion, probably caused by leakage via the seal contact and RC filtering should be considered.

## Introduction

Cell-attached patch-clamp recordings in voltage-clamp mode were pioneered for recording single ion channel activity and remain an unique technique (along with inside-out and outside-out) for exploring ion channel activity on the sub-millisecond time scale (Neher and Sakmann, 1976; Hamill et al., 1981). In addition, cell-attached voltage-clamp recordings have been implemented for recording and evoking action potentials in neurons (Chavas and Marty, 2003; Perkins, 2006; Alcami et al., 2012; Khalilov et al., 2015), estimation of the resting membrane potential (*E*_*m*_) from the reversal potential of the currents through potassium and NMDA channels (Khazipov et al., 1995; Verheugen et al., 1995, 1999; Fricker et al., 1999; Tyzio et al., 2003), and driving force acting on currents through GABA channels (Tyzio et al., 2006, 2008; Khazipov et al., 2008). An advantage of the cell-attached recording technique is that it allows access to neuronal functions without cell membrane rupture, thus avoiding an introduction of leakage conductance via the seal between the pipette and the membrane (Barry and Lynch, 1991; Tyzio et al., 2003) and without alteration in the intracellular medium caused by cell dialysis (Pusch and Neher, 1988). Performed in current-clamp mode, cell-attached recordings allow assessment of cell voltage including resting *E*_*m*_, action potentials, synaptic events and agonist responses (Fenwick et al., 1982; Hayar et al., 2004; Mason et al., 2005; Perkins, 2006; Kirmse et al., 2015). The rationale for cell-attached current-clamp (CA/CC) recordings is that with seal resistance (*R*_*seal*_) much greater than patch resistance (*R*_*patch*_), the voltage at the cell-attached patch-pipette should approach the *E*_*m*_ value. Simultaneous whole-cell (WC) and CA/CC recordings from electrically compact rat basophilic leukemia cells and megakaryocytes revealed a high degree of accuracy in measuring *E*_*m*_ using the CA/CC approach and the ability of this technique to monitor dynamic changes in membrane potential in these non-excitable cells (Mason et al., 2005). However, it has been noted that the accuracy of the measurement of the dynamic changes in membrane potentials maybe limited by the filtering of fast signals by the membrane patch RC filter (Fenwick et al., 1982; Mason et al., 2005; Perkins, 2006). However, the accuracy with which CA/CC recordings measure *E*_*m*_ and its dynamic changes in neurons has not been directly addressed.

Here, were explored this issue using concomitant CA and WC recordings of neocortical and hippocampal neurons in mouse brain slices. We found that CA/CC recordings are suitable for assessing steady-state membrane potential, synaptic activity, action potentials, depolarizing and hyperpolarizing actions of GABA, and cellular behavior during network-driven activity, but with allowance for possible errors compromising signal amplitude and kinetics, including leakage of the seal and RC filtering.

## Materials and methods

### Ethical approval

The animal experiments were carried out in compliance with the ARRIVE guidelines. Animal care and procedures were in accordance with EU Directive 2010/63/EU for animal experiments, and all animal-use protocols were approved by the French National Institute of Health and Medical Research (APAFIS #16992-2020070612319346 v2) and the Local Ethical Committee of Kazan Federal University (No24/22.09.2020).

### Brain slice preparation

C57BL/6 mice of both sexes aged from postnatal days [P] 5 to 60 were used. Animals were decapitated under isoflurane anesthesia (5%), the brain was rapidly removed and placed in ice-cold (2–5°C) slicing solution of the following composition (in mM): K-Gluconate 140, Na-Gluconate 15, NaCl 4, EGTA 0.2, D-AP5 50 μM and HEPES 10 (pH 7.4) (for cortical recordings) or NaCl, 125; NaHCO_3_, 25; KCl 2.5; NaH_2_PO_4_ 1,25; MgCl_2_, 1; CaCl_2_, 2; H D-glucose, 25 (for hippocampal recordings). Four hundred and fifty micrometer thick horizontal slices were cut using a PELCO easiSlicer™ vibratome (Ted Pella, Inc., Redding, CA, USA). Slices were first kept in oxygenated (95% O_2_-5% CO_2_) artificial cerebrospinal fluid (ACSF) of the following composition (in mM): NaCl 126, KCl 3.5, CaCl_2_ 2, MgCl_2_ 1.3, NaHCO_3_ 25, NaH_2_PO_4_ 1.2 and glucose 11 (pH 7.4) for 30 min at 32 ^o^C and then at room temperature (20–22 ^o^C) for at least 1 h before use.

### Electrophysiological recordings

**Figure 1:**
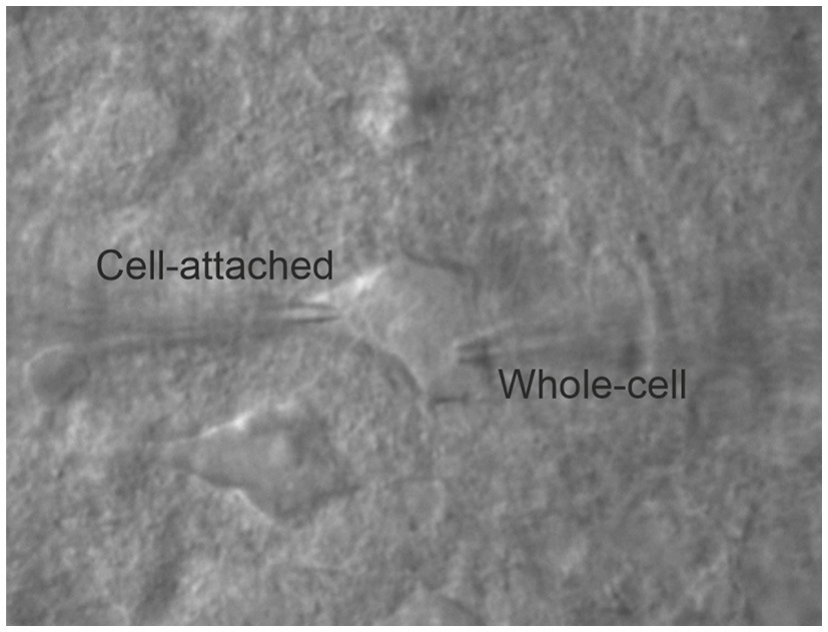
Dual whole-cell and cell-attached recordings from cortical neurons in brain slice. Differential interference contrast image of a L5 pyramidal cell in mouse somatosensory cortex. Cell-attached (left) and whole-cell patch electrodes (right) allow simultaneous recordings from the same neuron.

**Figure 2:**
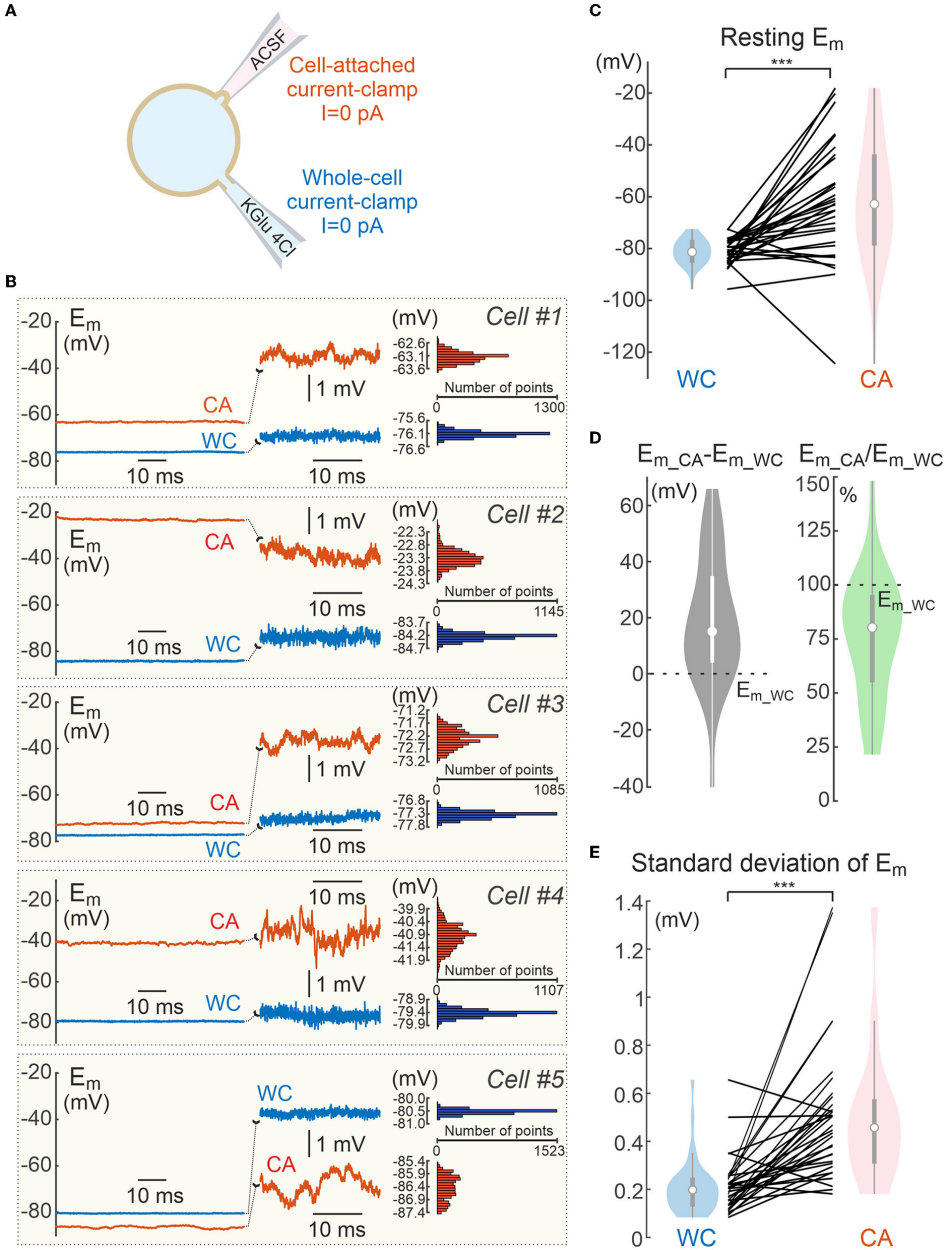
Resting membrane potential of L5 cortical neurons during dual cell-attached and whole-cell recordings in current-clamp mode. **(A)** Scheme of whole-cell and cell-attached recordings of the resting membrane potential. Null current (I = 0) is injected to either of electrodes. **(B)** Example dual recordings of the resting membrane potential (*E*_*m*_*rest*_) from five L5 pyramidal neurons in whole-cell (WC, blue traces) and cell-attached (CA, red traces) configurations in current-clamp mode with common *E*_*m*_ scale (left panels) and with expanded *E*_*m*_ scale (middle panels). The right panels show corresponding *E*_*m*_ all point histograms. **(C)** *E*_*m*_*rest*_ values measured in paired WC and CA recordings. Hereafter, each line represents paired WC and CA data from an individual neuron, violin plots show the probability density of the data at different values smoothed by a kernel density estimator, white dots show the median of the data, vertical gray bar indicates the interquartile range. **(D)** Difference between (left) and ratio of (right) CA and WC *E*_*m*_*rest*_ values. **(E)** Baseline noise assessed as standard deviation of *E*_*m*_ in CA and WC current-clamp recordings. **(C–E)** Pooled data from 32 L5 pyramidal cells of mouse somatosensory cortex and CA1 hippocampus (age P14–P30). ***P < 0.001.

**Figure 3:**
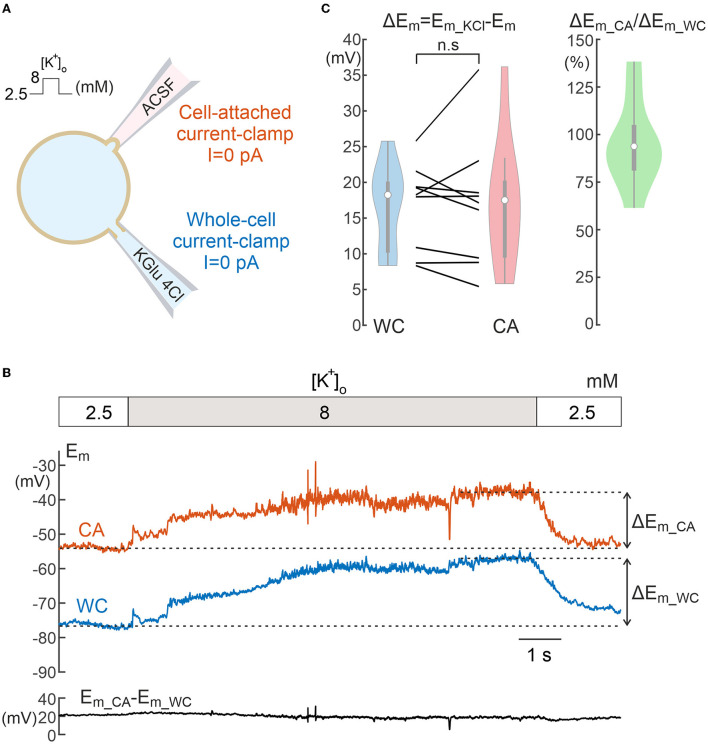
Comparison of neuronal depolarization induced by elevated potassium during cell-attached and whole-cell recordings. **(A)** Scheme of recordings. **(B)** Example of the response to an increase in [K^+^]_o_ from 2.5 to 8 mM in the bath solution during dual WC and CA recordings in a L5 pyramidal neuron. The dashed lines indicate the steady-state *E*_*m*_ levels used to calculate *E*_*m*_ depolarization during [K^+^]_o_ elevation. The trace below shows a difference between the *E*_*m*_ values in CA and WC throughout the experiment. **(C)** Neuronal depolarization in response to high potassium in paired WC and CA recordings (left) and corresponding CA/WC transfer coefficient (right). Pooled data from 9 L5 pyramidal cells of mouse somatosensory cortex. n.s, non-significant.

**Figure 4:**
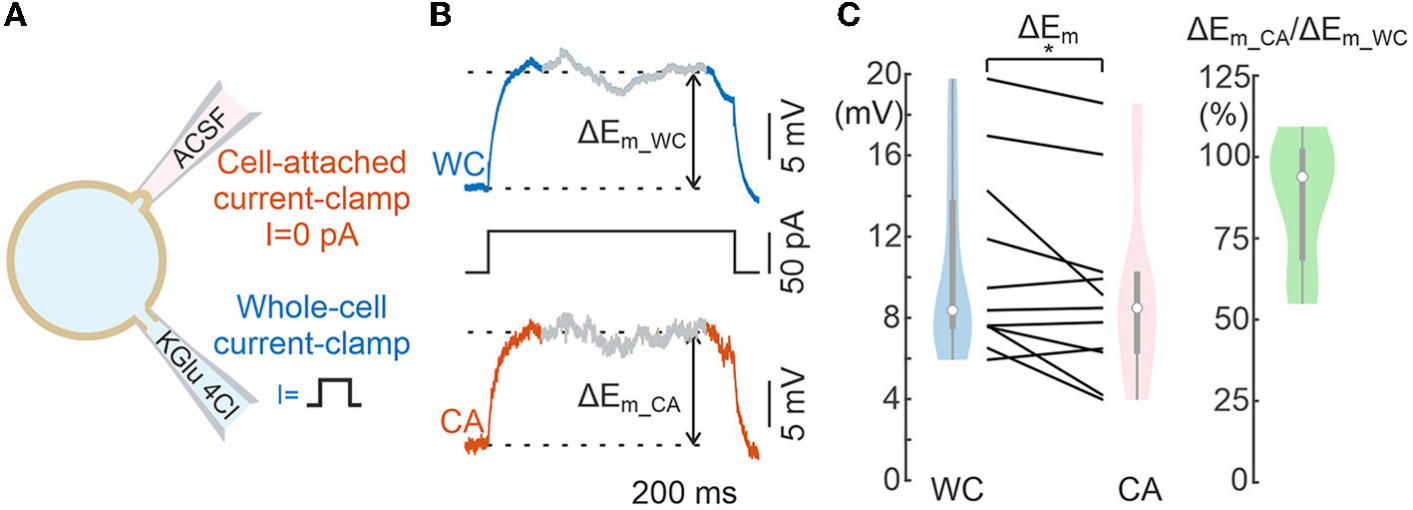
Comparison of voltage responses evoked by current steps during whole-cell and cell-attached recordings. **(A)** Scheme of recordings. **(B)** Example of the response to a current step applied to WC electrode during dual WC and CA recordings from a L5 pyramidal neuron. The section of the signal from which the response value was calculated is shaded. The dashed lines indicate *E*_*m*_ levels used to calculate the voltage response. **(C)** Neuronal depolarization in response to WC- current steps in paired WC and CA recordings. The left plot shows the absolute values and the right plot shows the corresponding CA/WC transfer coefficient. Pooled data from 11 L5 pyramidal cells of mouse somatosensory cortex. *P < 0.05.

**Figure 5:**
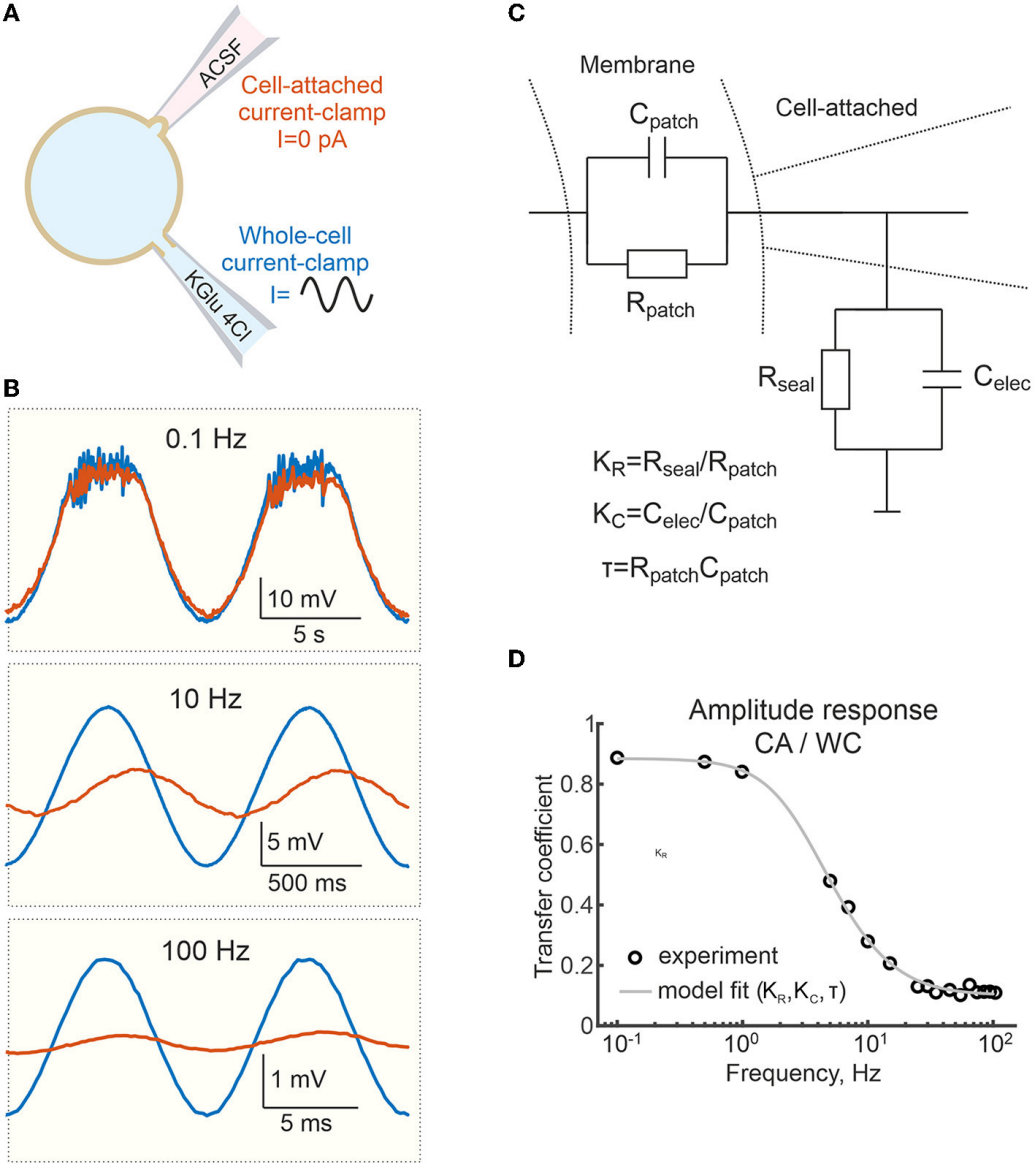
Frequency dependence of the voltage response to sinusoidal currents in cell-attached and whole-cell recordings. **(A)** Scheme of recordings. Sinusoidal currents of different frequencies are applied to the WC electrode. **(B)** Examples of responses to sinusoidal currents of different frequencies applied to WC electrode during dual WC and CA recordings from a L5 pyramidal neuron. **(C)** Electrical equivalent model of CA recordings. *C*_*patch*_ and *R*_*patch*_ are capacitance and resistance of the cell-attached membrane patch; *C*_*elec*_ and *R*_*seal*_ are capacitance of the patch pipette and seal resistance. **(D)** Transfer coefficient (amplitude response) during CA recordings relative to the WC response as a function of the frequency of the sinusoidal current applied to the WC- electrode. The circles are experimental data from a single cell, the gray line shows fit with a model on **(C)**.

**Figure 6:**
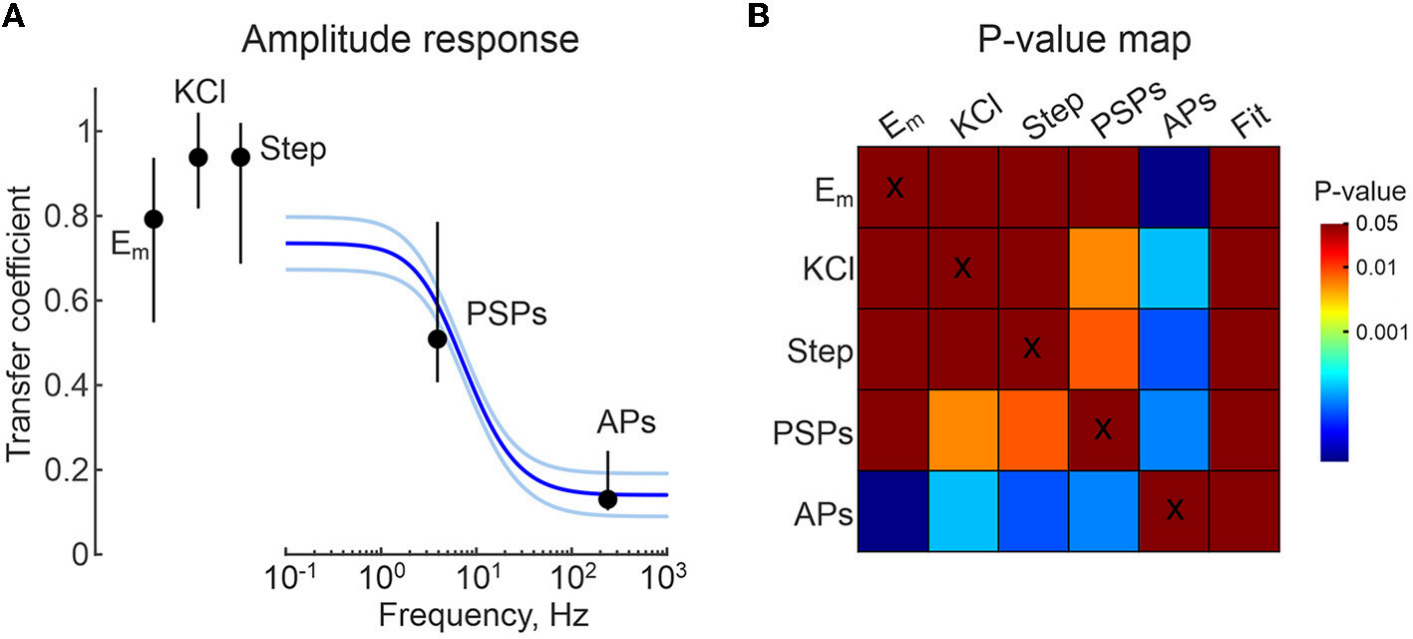
Group data on frequency dependence of CA/WC transfer coefficient. **(A)** Group data on the frequency dependence of the CA/WC amplitude transfer coefficient of sinusoidal signals obtained from 6 L5 neurons. The blue line shows the average fit, the iced blue lines show the standard errors. CA/WC transfer coefficients for *E*_*m*_*rest*_ values (from Figure 2), responses to high-potassium (from Figure 3) and current steps (from Figure 4), spontaneous postsynaptic potentials (from Figure 8) and APs (from Figure 7) are shown as median value ± interquartile range. **(B)** *P*-value map for comparison between all groups shown in **(A)**. The color of the cell at the row-column intersection shows the statistical significance (*p*-value) for the corresponding parameters. The last column shows the *p*-value for the corresponding comparison with the fitting results at the appropriate frequency (from the panel **A**). The dark red color corresponds to non-significant differences (*p* > 0.05), the other colors show different levels of significance of differences (*p* < 0.05) according to the color code (color bar on the right).

**Figure 7:**
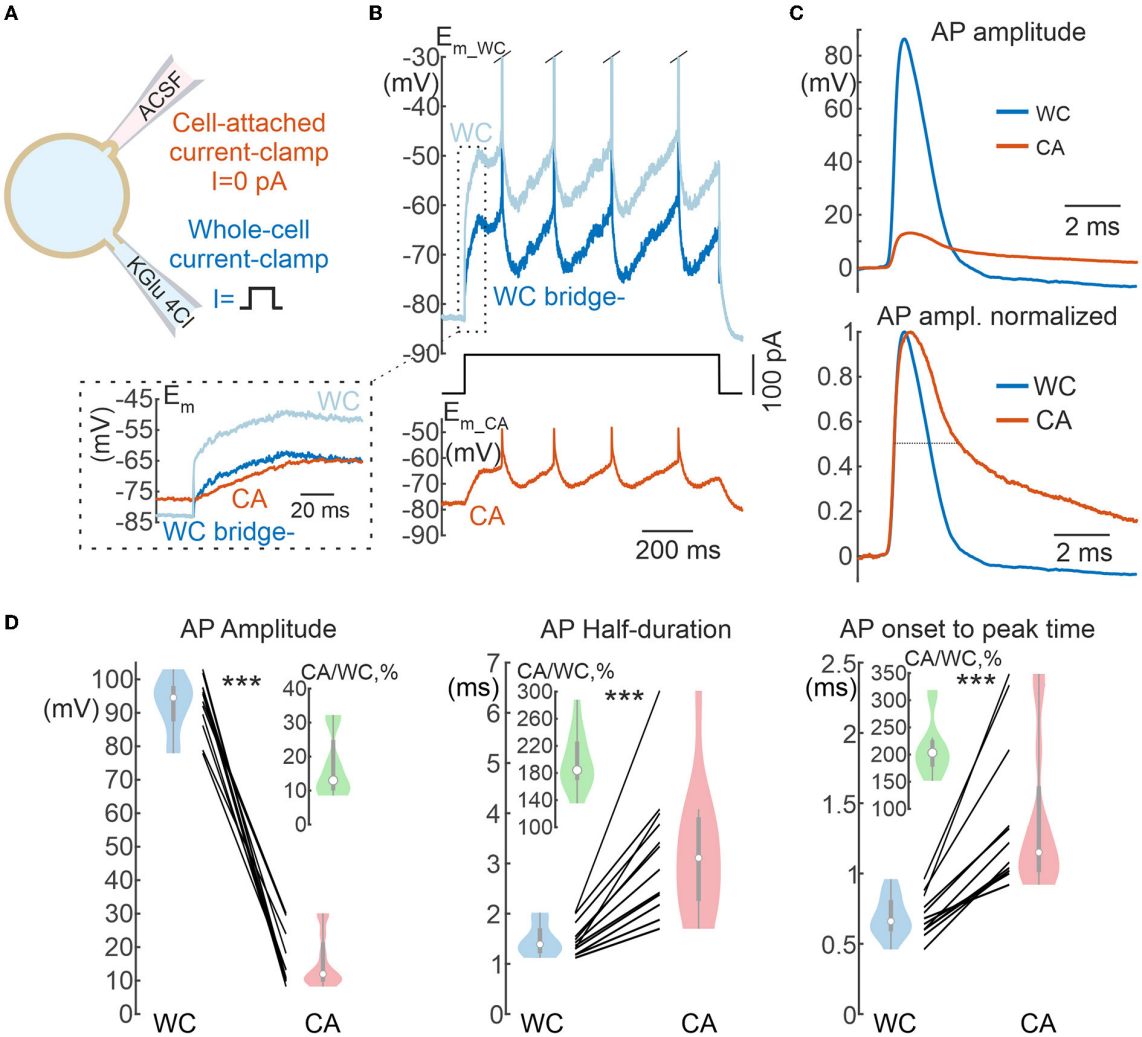
Action potentials in cell-attached and whole-cell recordings. **(A)** Scheme of recordings. APs are evoked by suprathreshold depolarizing current steps applied to the WC electrode. **(B)** Example of a response to a suprathreshold current step applied to the WC electrode during dual WC and CA recordings from a L5 pyramidal neuron. The ice-blue and blue traces show WC responses before (WC) and after (WC bridge -) bridge subtraction, respectively. Note that the bridge current is absent in the CA trace (inset). **(C)** APs in the CA and WC recordings with common voltage scale (top panel) and normalized to peak amplitude (bottom panel), dashed line is placed at half-amplitude. The resting membrane potential values were subtracted. **(D)** AP parameters in paired WC and CA recordings: AP amplitude (left), half-duration (middle), and time from onset to peak (right). The insets show in green the values of corresponding CA/WC transfer coefficients. Pooled data from 12 pyramidal cells in the L5 somatosensory cortex of the mouse. ***P < 0.001.

**Figure 8:**
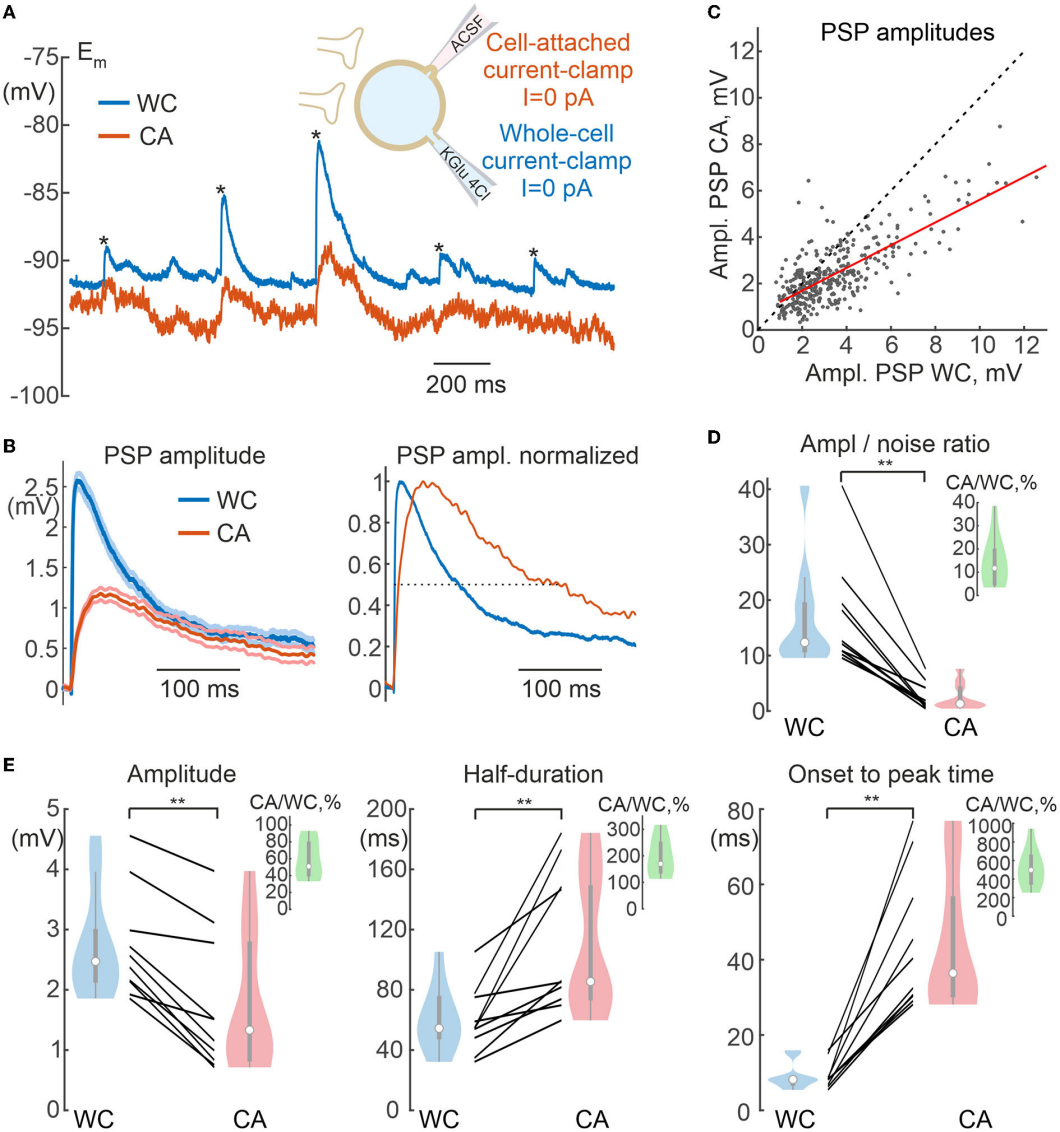
Spontaneous postsynaptic potentials in cell-attached and whole-cell recordings. **(A)** Scheme of recordings. Spontaneous postsynaptic potentials (sPSPs) are recorded using CA and WC electrodes from same neuron at resting membrane potential. WC electrode contains a low-chloride solution (4 mM), therefore most sPSPs are glutamatergic in nature. **(B)** Averaged sPSPs in WC and CA recordings with a common voltage scale (left panel) and normalized to peak amplitude (right panel), dashed line is placed at half-amplitude. The resting membrane potential values are subtracted. **(C)** Relationship between amplitudes of sPSPs in the WC and CA configurations for the cell shown in **(A)**. Each dot represents an individual sPSP. The red line shows a linear fit. **(D)** Amplitude to noise ratio for sPSPs in WC and CA recordings. The inset shows CA/WC transfer coefficient values. **(E)** Parameters of sPSPs in paired WC and CA recordings: amplitude of sPSPs (left), half-duration (middle), and time from onset to peak (right). The insets in green show the values of the respective CA/WC transfer coefficients. **(C,D)** Pooled data from 10 pyramidal cells in the L5 somatosensory cortex of the mouse. **P < 0.01.

**Figure 9:**
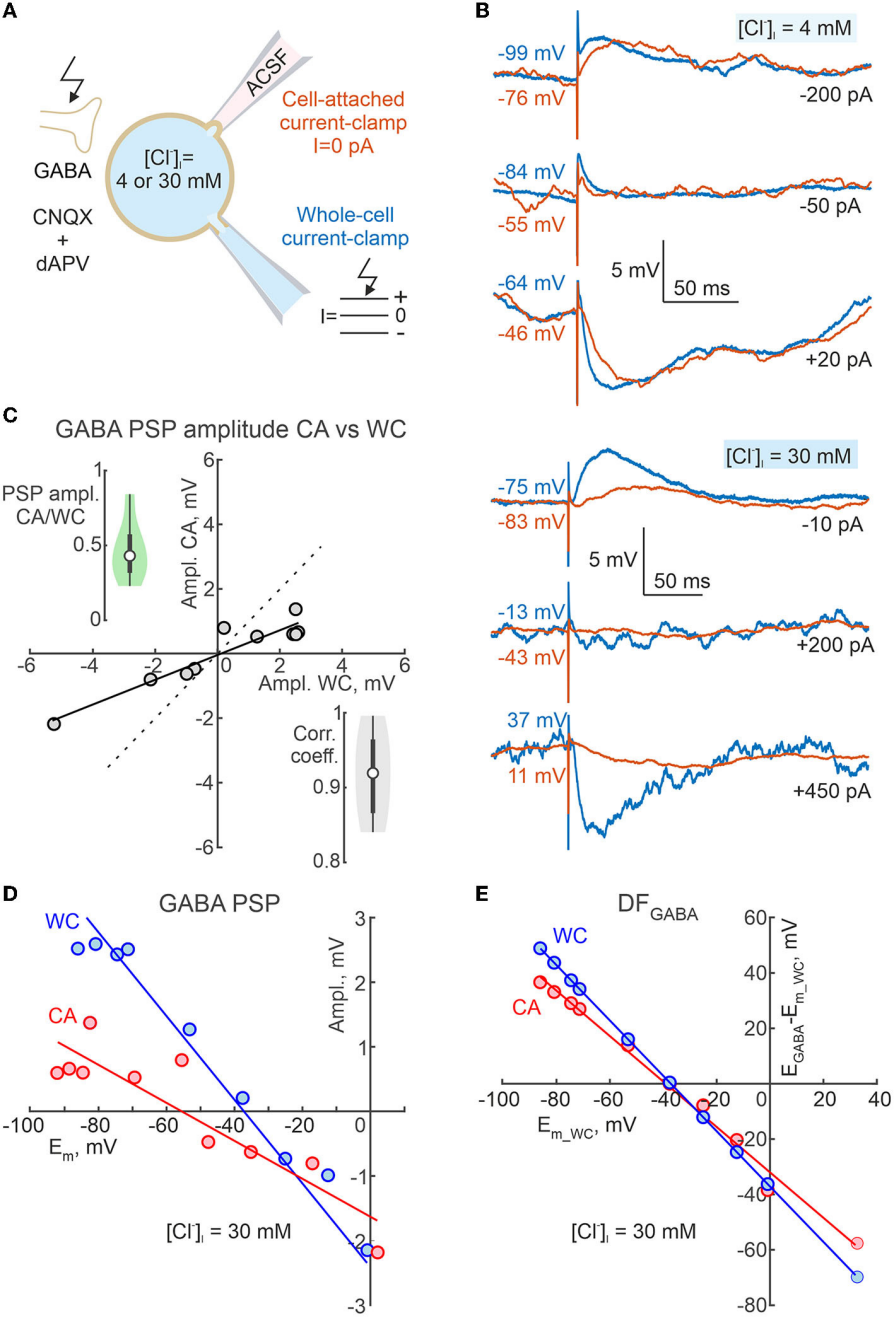
Dependence of the polarity of GABA-PSPs during cell-attached current clamp recordings on neuronal membrane potential and intracellular chloride. **(A)** Scheme of the experimental setup. Dual CA and WC current-clamp recordings of the pharmacologically isolated, electrically evoked GABA-PSPs were performed from adolescent CA1 pyramidal cells at different *E*_*m*_ values imposed by current injection into the WC electrode, and at various [Cl^−^]_i_ (4 mM and 30 mM) in the WC pipette solution. Ionotropic glutamate receptors were blocked by CNQX (10 μM) and d-APV (40 μM). **(B)** Examples of evoked GABA-PSPs during concomitant CA and WC recordings with [Cl^−^]_i_ = 4 mM (top traces) and 30 mM (bottom traces) in the WC pipette at different membrane potentials. **(C)** Relationships between amplitudes of evoked GABA-PSPs in WC and CA configurations for a cell shown on **(B)** with [Cl^−^]_i_ = 30 mM. The points represent the average of 10 GABA-PSP recorded at different *E*_*m*_*wc*_ values. Note that GABA-PSPs in WC and CA have similar polarity and that the conductance of GABA-PSPs in CA is smaller than in WC. The insets show group data on the CA/WC transfer coefficient of GABA-PSP amplitude (top left) and the correlation coefficient between GABA-PSP amplitude in CA and WC (bottom right) (*n* = 8 CA1 pyramidal cells from P15-19 mice). **(D)** Dependence of GABA-PSPs amplitude in WC and CA recordings on *E*_*m*_*wc*_ and *E*_*m*_*ca*_, respectively for a CA1 pyramidal neuron recorded with [Cl^−^]_i_ = 30 mM in the WC pipette solution. The *E*_*m*_ scale is the same for *E*_*m*_*wc*_ and *E*_*m*_*ca*_. **(E)** Dependence of *DF*_*GABA*_ relative *E*_*m*_*wc*_ for GABA-PSPs recorded in CA and WC for a cell shown on **(D)**.

**Figure 10:**
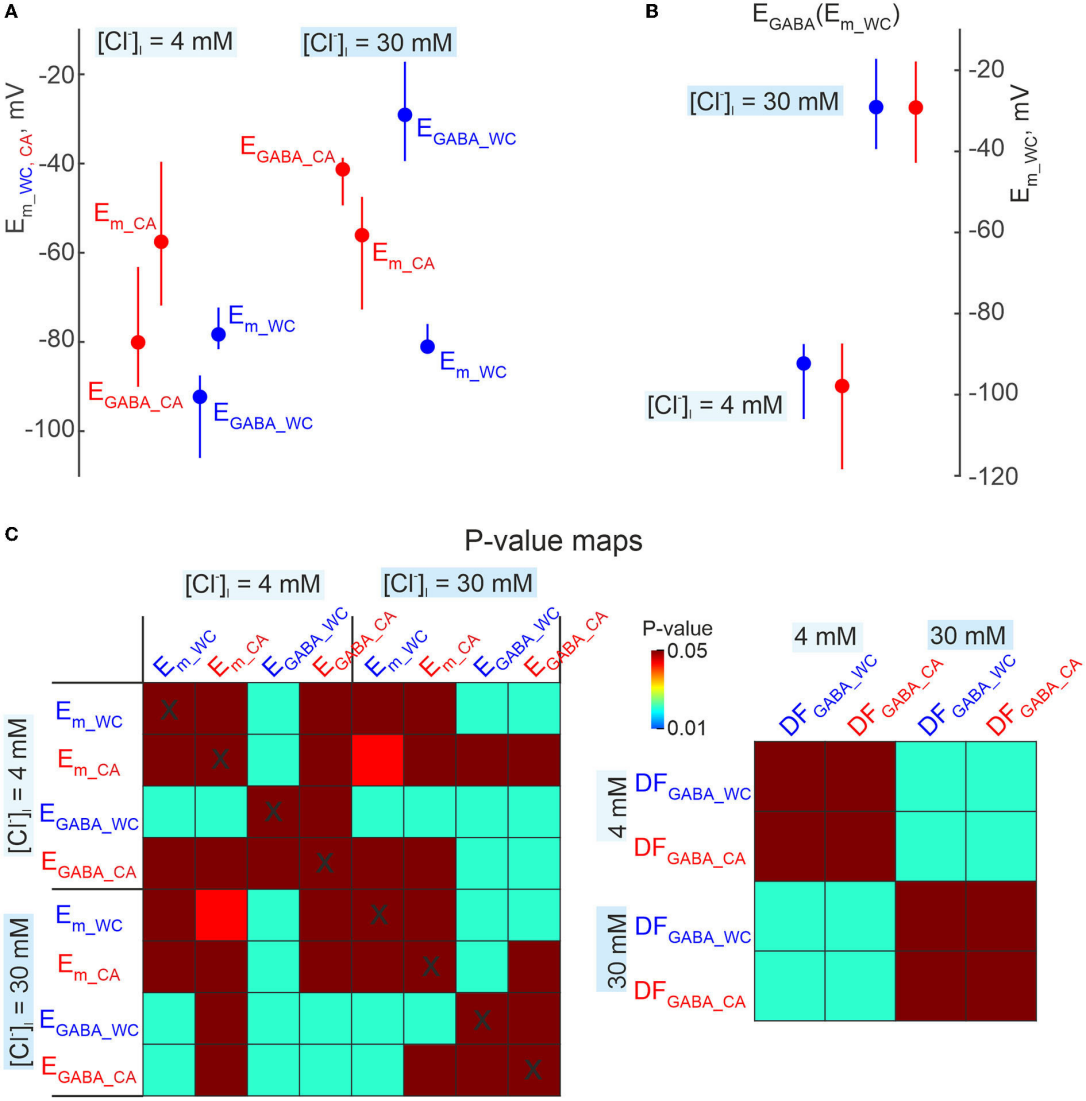
Comparisons of E_GABA_ derived from dual cell-attached and whole-cell recordings. **(A)** Summary plot of resting *E*_*m*_ and *E*_*GABA*_ obtained during CA and WC recordings of GABA-PSPs in CA1 pyramidal cells with [Cl^−^]_i_ = 4 mM and 30 mM in the WC pipette solution. Note that *E*_*GABA*_ values were calculated relative to *E*_*m*_ values in CA and WC, respectively. The difference in resting *E*_*m*_ values results in an apparent difference in *E*_*GABA*_ estimates in CA and WC recordings. **(B)** *E*_*GABA*_ values from the same dataset as in **(A)**, recalculated as a function of *E*_*m*_ in WC both for WC and CA data. This approach minimizes the error caused by a difference in *E*_*m*_ values in CA and WC, and *E*_*GABA*_ values in CA and WC show no difference at both [Cl^−^]_i_ = 4 mM and 30 mM. **(C)** Corresponding *p*-value maps for all parameters shown in **(A,B)**. The color of the cell at the row-column intersection shows the statistical significance (*p*-value) for the corresponding parameters. The dark red color corresponds to non-significant differences (*p* > 0.05), the other colors show different levels of significance of differences (*p* < 0.05) according to the color code (color bar on the right panel). **(A–C)** Pooled data from 8 pyramidal cells in the L5 somatosensory cortex of the mouse.

**Figure 11:**
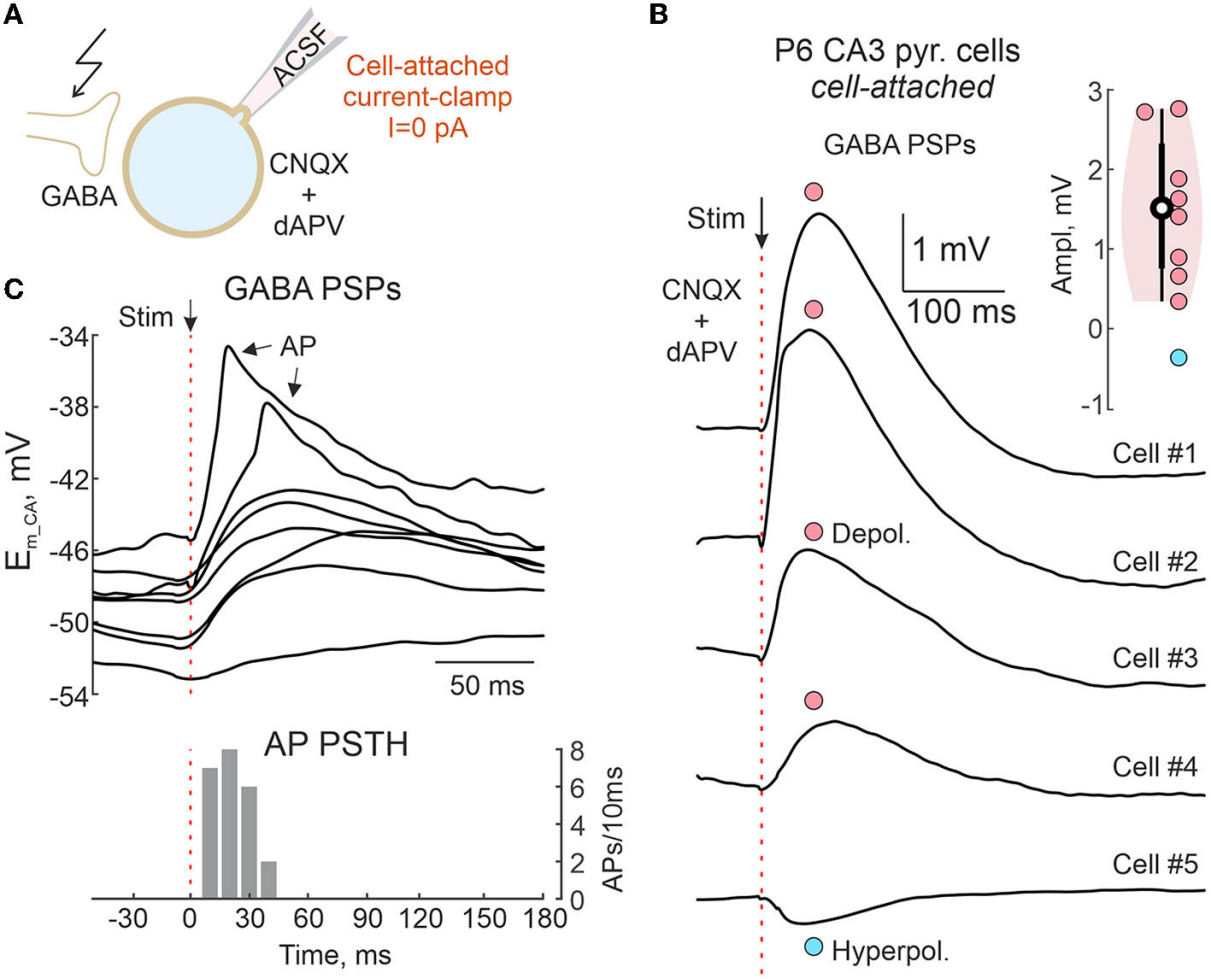
Cell-attached recordings of GABAergic postsynaptic responses in the neonatal hippocampus. **(A)** Scheme of the experimental setup. GABA-PSPs are evoked by electrical stimulation in the presence of the ionotropic glutamate antagonists CNQX (10 μM) and d-APV (40 μM) in a neonatal mouse hippocampal slice. Responses were recorded from CA3 pyramidal cells in CA/CC configuration without disturbance of intracellular chloride concentration. **(B)** Average evoked GABA-PSPs in five CA3 pyramidal cells, of which four show depolarizing responses (cells #1–4) and one (cell #5) shows a hyperpolarizing response. Inset shows group data on the amplitude of evoked GABA-PSPs recorded from 9 CA3 pyramidal cells of P6 mouse. **(C)** Example traces of evoked GABA-PSPs, some of which trigger APs. Below, a corresponding AP peristimulus time histogram.

**Figure 12:**
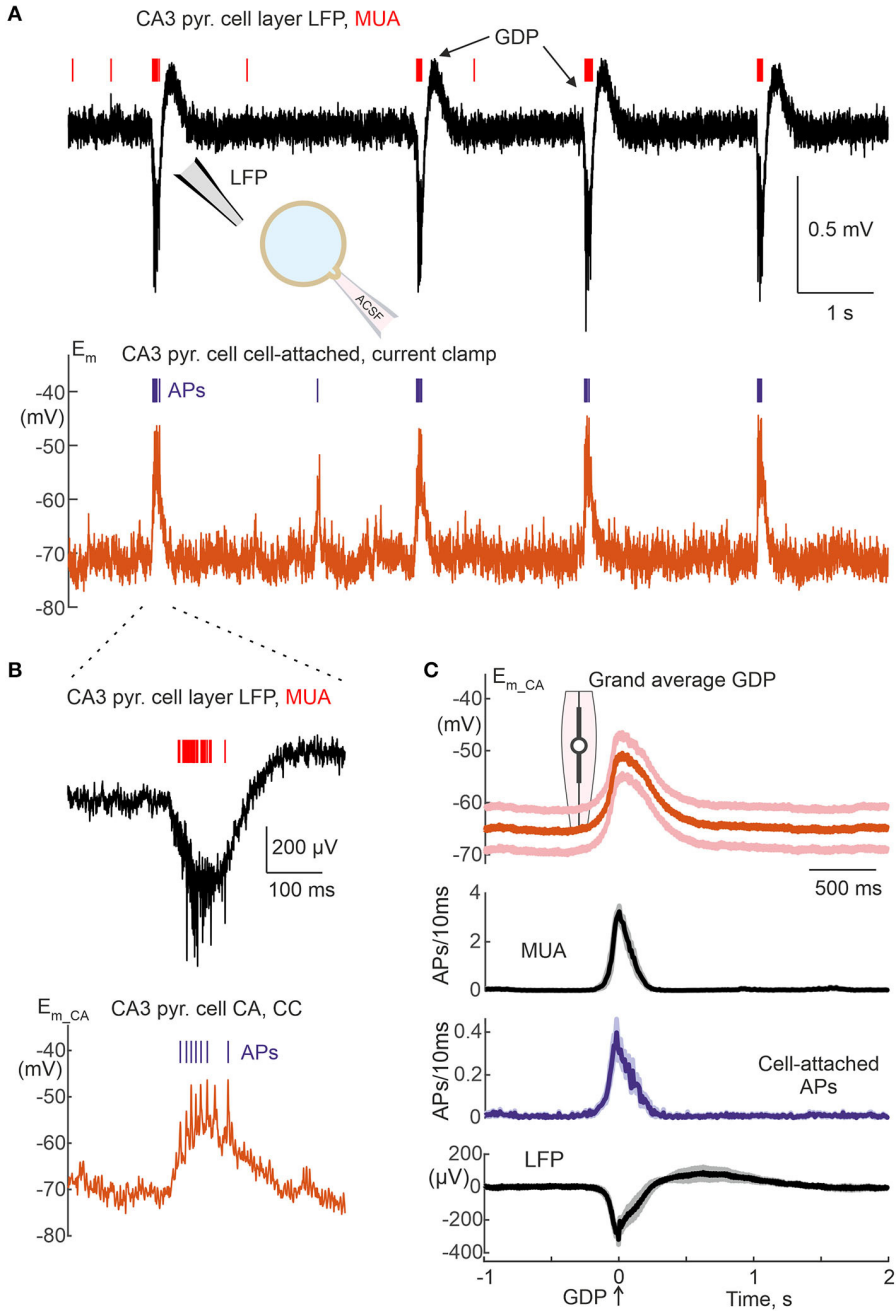
Cell-attached current-clamp recordings of Giant Depolarizing Potentials in the neonatal hippocampus. **(A)** Example recordings of local field potential (upper black trace) and MUA (red bars) from the CA3 pyramidal cell layer and CA/CC recordings from the CA3 pyramidal cell in a slice of neonatal (P5) mouse hippocampus showing spontaneous Giant Depolarizing Potentials (GDPs). **(B)** Example GDP from **(B)** on an expanded time scale. **(C)** Group data on average membrane potential changes and AP timing in CA3 pyramidal cells during CA/CC recordings, and extracellular MUA and LFP in the CA3 pyramidal cell layer during GDPs. The solid lines show the average, the shaded lines show ± SE. The violin plot shows the peak depolarization during GDPs. The peak negativity of the field GDP is taken as a time reference. Pooled data from 6 cells (37–92 GDPs/cell) from P5 mouse hippocampal slices.

**Figure 13:**
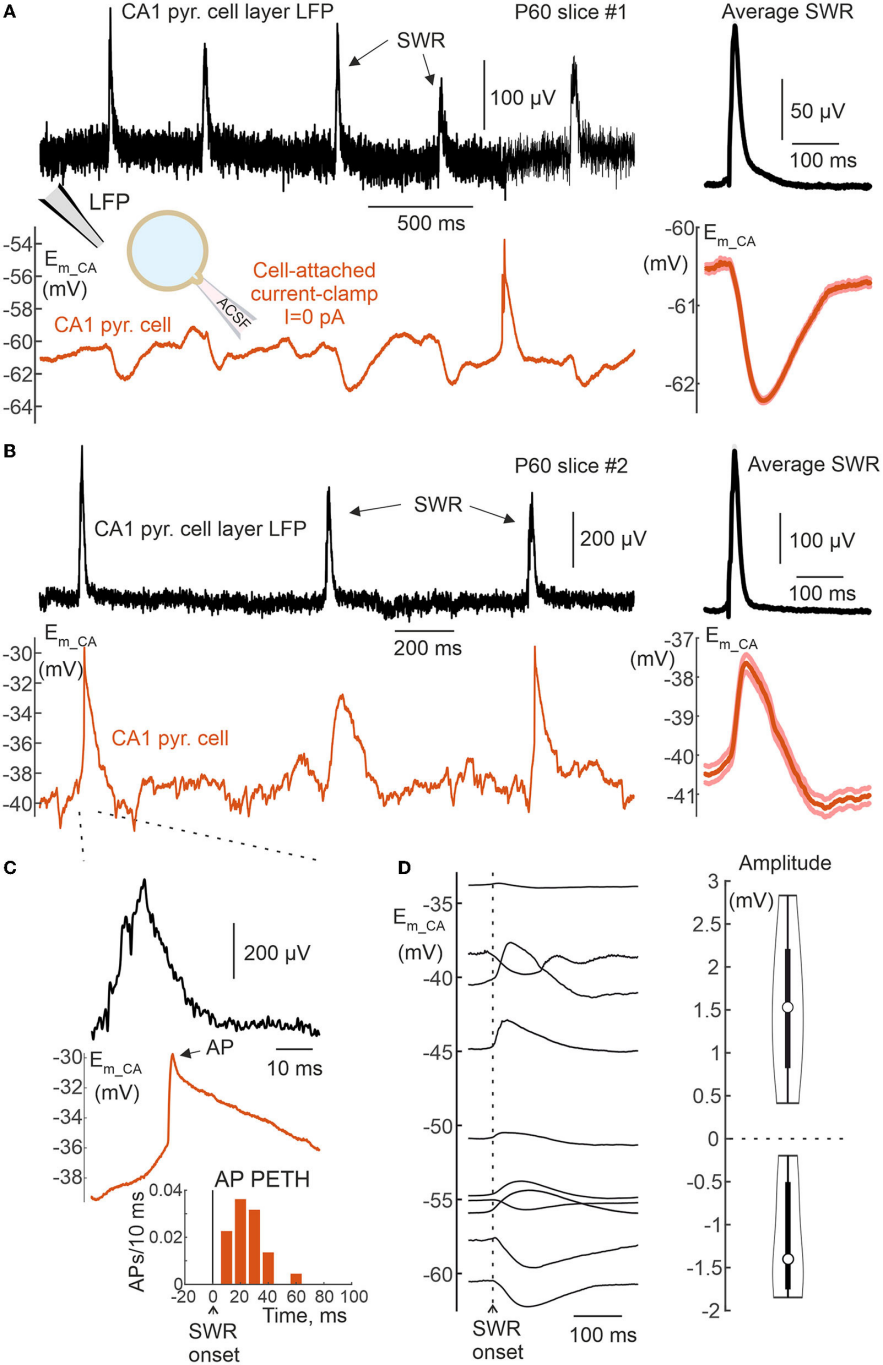
Cell-attached current-clamp recordings of Sharp Wave Ripples in the adult hippocampus. **(A,B)** Example recordings of the local field potential (upper black trace) from the CA1 pyramidal cell layer and CA/CC recordings from a CA1 pyramidal cell in a slice of the hippocampus of a 2-month-old mouse showing spontaneous Sharp Wave Ripples (SWRs) associated with neuronal hyperpolarization **(A)** and depolarization **(B)**. Averaged field and CA SWRs are shown on the right. **(C)** Example SWR from **(B)** on expanded time scale and corresponding peri-SWR AP histogram. **(D)** Average voltage change during SWR in 10 CA1 pyramidal cells, five of which show depolarization and another five hyperpolarization. On the right, the corresponding group data on the amplitude of *E*_*m*_ change during SWR recorded from 10 CA1 pyramidal cells of the 2-month-old mice.

For recordings, slices were placed into a submerged chamber and superfused with oxygenated ACSF at 30–32°C at a flow rate of 2–3 ml/min. Individual neurons were identified using a vertical microscope (BX-51 WI; Olympus, Japan) at 40× magnification using infrared differential interference contrast microscopy (IR-DIC). Recordings were made from neurons of the neocortex (Figures 1–8), and CA1 (Figures 2, 9, 10, 13) and CA3 (Figures 11, 12) regions of the hippocampus. Whole-cell recordings were performed using electrodes prepared from borosilicate glass with resistance of 5–7 MOhm when filled with the pipette solution of the following composition (in mM): (1) K-Gluconate 144, KCl 4, MgATP 4, NaGTP 0.3, HEPES 10, phosphocreatine, or (2) K-Gluconate 105, KCl 30, MgATP 4, NaGTP 0.3, HEPES 10, phosphocreatine 10 (pH 7.26). For solutions 1 and 2, the equilibrium Nernst potentials for chloride were −92 and −39 mV, respectively. Cell-attached recordings were performed using electrodes of 10–17 MOhm when filled with the pipette solution containing (in mM): (3) NaCl 125; NaHCO_3_ 25; KCl 2.5; NaH_2_PO_4_ 1.25; MgCl_2_ 1; CaCl_2_ 2; H D-glucose, 25. *E*_*m*_ values obtained during WC recordings were corrected for the liquid junction potential of 16 mV and 12.5 mV for the pipette solutions containing 4 Cl^−^ and 30 Cl^−^, respectively. Patch-clamp signals were recorded and digitized using EPC8 (HEKA electronics, Germany) with a sampling rate 50 kHz. For local field potential recordings, single-wire 50 μm tungsten electrodes or 16-channel iridium silicone probes were used. Signals were recorded and digitized using Open Ephys (Cambridge, Massachusetts) at a sampling rate of 30 kHz. The electrode of the silicone probe closest to the recorded cell was used for the analysis. GABAergic postsynaptic potentials were evoked by electrical stimulation in stratum radiatum of hippocampus in the presence of CNQX (10 μM) and APV (40 μM).

### Data analysis

Data were analyzed using custom-written procedures in Matlab (MathWorks, Inc., Natick, MA, USA). Preprocessing, preliminary analysis and review of the data were performed using ExpressAnalysis and Eview tools (https://github.com/AndreyZakharovExp). In the recordings with current injection through the whole-cell pipette, off-line bridge compensation was made by subtracting a rectangular-shaped potential, at the boundaries of which the first derivative of the whole-cell potential exceeds the empirically determined threshold (30% of the derivative peak value during the ON-phase of the step). In the recordings with sinusoidal current injection, *E*_*m*_ signals recorded in CA and WC configurations were fit by sinusoids, from which the CA/WC amplitude ratio was estimated. To fit the experimental values of the amplitude response (*AR*), we used the function calculated from the circuit diagram of the model (Figure 5C):


(1) $$AR\left(f\right)=\frac{\sqrt{{\left(2\cdot \pi \cdot K_{R}\cdot \tau \cdot f\right)}^{2}+K_{R}^{2}}}{\sqrt{{\left(2\cdot \pi \cdot (K_{C}+1)\cdot K_{R}\cdot \tau \cdot f\right)}^{2}+{\left(K_{R}+1\right)}^{2}}}$$

Where *K*_*R*_ = *R*_*seal*_/*R*_*patch*_, *K*_*C*_ = *C*_*elec*_/*C*_*patch*_, *τ* = *R*_*patch*_· *C*_*patch*_.

The amplitude of AP and PSP was calculated as the maximum value after subtraction of the mean *E*_*m*_ value in the interval of 1 and 5 ms before onset. APs in WC recordings were detected as events exceeding a threshold of 40 mV above the resting potential and spontaneous PSPs were detected as events exceeding the threshold of 1 mV/ms on the first derivative of the *E*_*m*_ signal. The onset of events was determined at a threshold of 10 mV/ms (for APs) or 0.1 mV/ms (for PSPs). The amplitude-to-noise ratio of the PSPs was calculated as average amplitude of the PSP divided by the standard deviation of the quietest 1 s interval in the recording. The amplitude of evoked GABA-PSPs was calculated as the maximum signal deflection reached within 200 ms after the stimulus in the average data, subtracting the corresponding *E*_*m*_ median baseline value within 200 ms before the stimulus. Sharp-wave ripples and Giant Depolarizing Potentials were detected from the 1–100 Hz band passed local field potential signal at the threshold of 3 standard deviations of the quietest 100 s episode in the recording. APs in CA recordings were detected from the first derivative of *E*_*m*_ at a threshold of 1 mV/ms. Extracellular multiple unit activity was detected from 300 to 2,500 Hz band passed local field potential signal at a threshold of 3 standard deviation of the quietest 100 s episode in the recording. Dominant frequency of APs and PSPs was calculated on the assumption that the half-width of the event is 1/3 of the period.

### Statistical analysis

Statistical analysis was based on the non-parametric Wilcoxon signed rank and rank sum test (for paired and independent samples), or Kruskal-Wallis test with the significance level set at *p* < 0.05. Results are given as median [25th percentile; 75th percentile].

## Results

### Resting membrane potential

In the present study, we estimated the accuracy of CA/CC recordings of the membrane potential of cortical neurons in brain slices using dual CA and WC recordings from the same neurons (Figure 1). First, we compared resting membrane potential (*E*_*m*_) values measured with CA and WC electrodes when zero current was injected into either electrode (Figure 2A). Figure 2B shows example traces of WC and CA recordings from five L5 neurons of mouse somatosensory cortex. *E*_*m*_ values obtained during WC recordings (*E*_*m*_*wc*_) were −81.3 mV [-84.6 ÷ −77.3] mV (median [25th percentile 75th percentile]), range −95.7 to −72.5 mV (*n* = 32 cells) which is close to previously reported values (Gulledge and Stuart, 2003). *E*_*m*_ values obtained during CA recordings (*E*_*m*_*ca*_) were on average more depolarized than *E*_*m*_*wc*_ values and showed higher variability, reaching −62.8 [−78.1 ÷ −44.3] mV, range −124.5 to −18.0 mV (Figure 2C; *n* = 32 cells, *p* < 0.05, Wilcoxon Signed Rank Test). It should be noted that, in general, *E*_*m*_ values were more frequently depolarized in CA relative to WC, but *E*_*m*_*ca*_ values more negative than *E*_*m*_*wc*_ were also observed. At the group level, the difference between *E*_*m*_ values in CA and WC was 16.0 [5.4 35.4] mV, range −39.4 to 66.9 mV (*n* = 32 cells). Accordingly, the *E*_*m*_ transfer coefficient, measured as the ratio *E*_*m*_*ca*_/*E*_*m*_*wc*_, was close to 0.8 and showed great variability (Figure 2D). Furthermore, CA records were characterized by larger baseline fluctuations (Figure 2B), with the standard deviation of *E*_*m*_ from the mean being more than twice as high (Figure 2E). Thus, CA recordings provided more variable and, on average, more depolarized *E*_*m*_ estimates compared to those obtained using WC recordings.

### High potassium-induced depolarization

We further explored the responses evoked by bath application of high-potassium solution using CA and WC recordings from L5 neurons (Figure 3A). Increasing [K^+^]_o_ from control levels of 2.5 to 8 mM induced a slowly developing neuronal depolarization and neuronal firing in both WC and CA recordings (Figure 3B, note that APs are suppressed by <1 Hz low-pass median filtering for clarity). Despite any difference in initial resting *E*_*m*_ values during WC and CA recordings, the amplitude of depolarizing *E*_*m*_ shifts induced by high potassium was similar in CA and WC recordings, with the average CA/WC transfer coefficient close to 0.95 (Figure 3C; *n* = 9, *P* > 0.05, Wilcoxon Signed Rank Test).

### Response to current steps

We next examined the voltage responses elicited by subthreshold square pulse current steps of 50 pA and 1 s duration injected into the cell via the WC patch pipette (Figure 4A). Figure 4B shows exemplary responses to current steps in WC and CA. The magnitude of *E*_*m*_ shifts during current steps was similar for CA and WC recordings, and analysis of group data revealed significant difference between CA and WC responses, and the amplitude of CA responses tended to be smaller than that of WC responses, with a mean CA/WC transfer coefficient of nearly 0.9 (Figure 4C; *n* = 11 L5 pyramidal cells; *P* > 0.05, Wilcoxon Signed Rank Test).

### Responses to sinusoidal currents

The above results show that CA recordings estimate slow *E*_*m*_ changes in cortical neurons relatively reliably. Next, we examined the accuracy of cell-attached *E*_*m*_ measurements of voltage signals evoked by sinusoidal currents of different frequencies (from 0.1 to 100 Hz) injected into L5 pyramidal cells via the WC pipette (Figure 5A). As shown in Figure 5B, the voltage responses recorded in the CA configuration were similar to the WC response at low (0.1 Hz) frequency of the sinusoidal current. However, the amplitude of the CA voltage responses decreased more and more compared to the WC responses with an increase of the sinusoidal current frequency to 10 and 100 Hz. Figure 5D shows corresponding WC-to-CA transfer coefficient estimated as the ratio of the oscillation amplitude in CA to WC recordings as a function of sinusoidal current frequency in the range 0.1 to 100 Hz. Note that for this neuron, the transfer coefficient approaches 0.9 at a frequency <1 Hz. At higher frequencies, CA/WC transfer coefficient gradually decreases and approaches the value of 0.1 at frequencies >10 Hz.

We fitted these data with an electrical equivalence model for CA/CC recordings that takes into account two RC chains made by the cell membrane patch under the electrode (*R*_*patch*_ and *C*_*patch*_) and another by the electrode capacitance (*C*_*elec*_) and the seal resistance (*R*_*seal*_) (Figure 5C) (Fenwick et al., 1982; Tyzio et al., 2003; Mason et al., 2005; Perkins, 2006). In the low frequency range (from 0 to ~1 Hz) this filter mainly works as a voltage divider, with the transfer coefficient determined by the ratio *R*_*seal*_/(*R*_*seal*_+*R*_*patch*_). In the high-frequency range (above 100 Hz), the filter mainly works as a capacitive voltage divider and the amplitude ratio is limited by the ratio *C*_*patch*_/(*C*_*patch*_+*C*_*elec*_). The experimental data for amplitude response was fitted using function (1) (see Methods). The results of the fitting are shown as a gray line in Figure 5D. For *C*_*elec*_ of 7 pF, the best fit of the model to the experimental data for these recordings gave values of *C*_*patch*_ = 0.8 pF, *R*_*seal*_=55.6 GOhm, *R*_*patch*_=7.3 GOhm. The results of a similar analysis in a group of six L5 pyramidal cells are shown in Figure 6A, with estimates of *C*_*patch*_ = 0.8 [0.4 1.6] pF, *R*_*seal*_ = 15.4 [8.2 23.6] GOhm, *R*_*patch*_=4.9 [4.8 5.1] GOhm. The theoretical transfer coefficient approached values of 0.78 [0.63 0.85] at 0 Hz, which was close to the attenuation level of constant (resting *E*_*m*_) and slow (responses to current steps and high-potassium) signals in CA mode (Figures 6A,B). Model data also agreed with the experimental results on the degree of suppression of the AP and PSP amplitude in the corresponding frequency ranges (Figures 6A,B and see below).

### Action potentials and synaptic potentials

We further investigated the properties of fast physiological signals, action potentials (APs) and postsynaptic potentials (PSPs), during current-clamp CA recordings. L5 pyramidal cells were recorded with WC and CA electrodes, and APs were evoked in these neurons by suprathreshold depolarizing current steps at the WC electrode. Figure 7B shows examples of APs recorded in WC and CA configurations. Both the amplitude and the time course of the APs were severely distorted in CA recordings (Figure 7C). AP amplitude was reduced almost tenfold in CA recordings compared with WC recordings (CA/WC transfer coefficient: 0.13 [0.10 0.24], *n* = 12), whereas AP half-duration and onset-to-peak time were almost 2-fold longer in CA recordings (Figure 7D). Extremely low values of the CA/WC transfer coefficient of AP amplitude corresponded to the frequency response characteristics of the sinusoidal evoked responses at the dominant frequency of the AP waveform (240 [200 270]) Hz; *n* = 12) (Figures 6A,B).

Spontaneous postsynaptic potentials (sPSPs) were recorded from L5 pyramidal cells with WC and CA electrodes at the resting membrane potential. Since a WC pipette solution with low (4 mM) chloride content was used in these recordings and the GABAergic postsynaptic potentials reverse near the resting membrane potential (see below), most of the sPSPs were probably glutamatergic in nature. Figure 8A shows exemplary traces of sPSPs in CA and WC recordings. It is noteworthy that sPSPs were less visible in CA than WC recordings due to the higher baseline noise (see also Figures 1B,D) and the amplitude of sPSPs was smaller, resulting in almost 10-fold lower signal-to-noise ratio in CA mode (Figure 8D). Therefore, the sPSPs were detected from the WC recordings and compared with the corresponding voltage traces from the CA recordings. As shown by the average trace of sPSP in a L5 pyramidal neuron in Figure 8B, the amplitude of the CA-sPSPs was almost half of that of the WC-sPSPs (see also Figure 8C), and their time course was slowed down. At the same time there was a high correlation between the amplitudes of CA-sPSPs and WC-sPSPs (Figure 8C). At the population level (*n* = 10 L5 pyramidal cells), CA-sPSPs were 2-fold smaller in amplitude than WC-sPSPs (CA/WC transfer coefficient: 0.51 [0.41 0.79], *n* = 10), and their half-duration and rise time were significantly prolonged (Figure 8E). As with APs, the CA/WC transfer coefficient of the sPSPs amplitude corresponded to the frequency response characteristics of sinusoidal evoked responses at the dominant frequency of the sPSPs waveform (3.9 [2.2 4.5] Hz; *n* = 10) (Figures 6A,B).

### Dependence of GABAergic responses on membrane potential and intracellular chloride

In addition, we wanted to know how reliable CA recordings are for assessing the polarity (depolarising or hyperpolarising) of GABAergic responses. To this end, we examined the dependence of the polarity of GABA-PSPs in dual CA and WC current-clamp recordings of GABA-PSPs on membrane potential and intracellular chloride (Figure 9A). GABA-PSPs were evoked in CA1 pyramidal cells of juvenile (2–3 weeks old) mice by electrical stimulation of the stratum radiatum in the presence of the ionotropic glutamate receptors antagonists CNQX (10 μM) and d-APV (40 μM). Figure 9B shows examples of evoked GABA-PSPs during simultaneous CA and WC recordings with [Cl^−^]_i_ = 4 and 30 mM in the WC pipette at different membrane potentials. Note that despite the different *E*_*m*_ values in CA and WC, the polarity of GABA-PSPs in both depolarizing and hyperpolarizing directions, as well as their reversal, were similar in WC and CA recordings. While the amplitudes of GABA-PSPs in the CA and WC recordings were highly correlated with a correlation coefficient >0.9, their amplitude in the CA recordings was on average twice as small as in the WC recordings (Figure 9C; *n* = 8 cells, *p* < 0.05; Wilcoxon Signed Rank test). As expected, a difference between resting *E*_*m*_ values in WC and CA recordings strongly biased GABA-PSPs reversal potential (*E*_*GABA*_) estimates from the relationships of GABA-PSPs amplitude from *E*_*m*_*ca*_ and *E*_*m*_*wc*_ (Figure 9D). However, both the *E*_*GABA*_ values and the GABA-driving force (*DF*_*GABA*_ = *E*_*GABA*_ - *E*_*m*_) calculated from the *E*_*m*_*wc*_ values agreed between the CA and WC recordings and showed a similar dependence on [Cl^−^]_i_ (Figures 9E, 10).

### Polarity of GABAergic responses in the neonatal hippocampus

The above results suggest that CA/CC recordings reliably report the polarity of GABAergic responses. We next tested this approach to characterize GABAergic responses in neonatal neurons from hippocampal slices, where depolarizing effects of GABA are well-documented (Ben-Ari et al., 1989; Ben Ari et al., 2007; Watanabe and Fukuda, 2015; Kirmse et al., 2018). Electrically evoked pharmacologically isolated GABA-PSPs were recorded from CA1 pyramidal neurons of neonatal (P6) mouse hippocampal slices in CA/CC configuration (Figure 11A). In contrast to the above experiments, no concomitant WC recordings were made to keep [Cl^−^]_i_ intact. Depolarizing GABA-PSPs with an average amplitude of +1.5 mV were recorded in eight cells under these conditions, and only one neuron showed slightly hyperpolarizing GABA-PSPs (Figure 11B). Depolarizing responses eventually triggered APs with delays similar to those reported with extracellular recordings (Figure 11C) (Valeeva et al., 2010).

### Giant depolarizing potentials

Neuronal activity in hippocampal slices of neonatal rodents is characterized by recurrent Giant Depolarizing Potentials (GDPs), the generation of which is closely linked to the depolarizing effect of GABA on immature neurons (Ben-Ari et al., 1989; Garaschuk et al., 1998; Menendez de la Prida et al., 1998; Ben Ari et al., 2007; Griguoli and Cherubini, 2017; Cossart and Khazipov, 2022). Since GABAergic conductance dominates during GDPs, non-invasive CA/CC recordings can be potentially useful for GDPs assessment. To this end, we performed CA/CC recordings of CA3 pyramidal cells from hippocampal slices of neonatal (P5) mice. Extracellular local field potential (LFP) and multiple unit activity (MUA) recordings from the CA3 pyramidal cell layer near the CA recordings were used to detect network-driven field GDPs (Figures 12A,B). We found that the GDPs and APs in the CA3 pyramidal cells were highly synchronized with field GDPs and MUA, and similar behavior was observed in all 6 neurons studied in this series, as shown by the grand average GDPs analysis in Figure 12C. During GDPs, CA3 pyramidal cells depolarized from resting *E*_*m*_*ca*_ −62.1 [−71.7 −57.9] mV to −49.0 [−55.4 −41.3] mV (i.e., by 16.3 [14.6 17.5] mV; *n* = 6 cells), which is close to GDP values reported in CA3 pyramidal cells using gramicidin perforated patch recordings (Khalilov et al., 2015) (Figure 12C). Maximal neuronal firing occurred at the peak of depolarization during GDPs, which coincided with MUA peak during extracellular recordings.

### Hippocampal sharp waves

Adult rodent hippocampal slices show recurrent bursts of activity that share a number of similar features with Sharp Wave Ripples (SWRs) *in vivo* (Maier et al., 2003; Behrens et al., 2005; Hajos et al., 2009; Buzsaki, 2015; Norimoto et al., 2018). Interestingly, the behavior of CA1 pyramidal cells during SWRs is variable *in vitro*, with different cells showing depolarization, hyperpolarization or mixed responses during SWRs (Maier et al., 2003). Here, we approached the behavior of CA1 cells during SWRs by using CA/CC recordings from hippocampal slices of 2-month-old mice, using a similar experimental approach as for GDPs in the previous section. Unlike GDPs, SWRs were associated with positive LFP deflections in the CA1 pyramidal cell layer, and these were used for SWR detection (Figures 13A,B). Consistent with the results of a previous study using intracellular recordings (Maier et al., 2003), we found that half of the CA1 neurons (5 of 10) were hyperpolarized during SWRs (Figure 13A), while another half of the CA1 neurons (also 5 of 10) were depolarized during SWRs. Finally, in the case of depolarizations, the neurons also fired APs during SWRs (Figure 13C). Resting *E*_*m*_ was highly variable in this dataset, but we found no significant correlation between *E*_*m*_ and voltage response in CA1 neurons during SWRs, which average circa +1.5 mV and −1.5 mV in cells with depolarizing and hyperpolarizing SWR-driven responses, respectively (Figure 13D).

## Discussion

The main aim of the present study was to determine the accuracy of the measurement of resting membrane potential and its dynamic changes in CA/CC recordings by comparing signals obtained in dual CA and WC recordings from the same neurons. Our overall conclusion is that CA/CC measurements are similar to WC recordings, have several advantages, including lack of cell dialysis, but also introduce several sources of error that should be considered when interpreting data obtained with CA/CC recordings.

### Resting membrane potential

We found that the *E*_*m*_*rest*_ values obtained in CA/CC recordings are typically slightly more depolarized compared to WC-values (on average, by 19 mV). The depolarized *E*_*m*_*rest*_ values in CA/CC are consistent with theoretical model of CA/CC recordings, according to which *E*_*m*_*rest*_ in CA should be more depolarized than *E*_*m*_*rest*_ in WC, and its value critically depends on the *R*_*seal*_ / *R*_*patch*_ ratio. When *R*_*seal*_ >> *R*_*patch*_, *E*_*m*_*rest*_ in CA approaches the true *E*_*m*_*rest*_ value. However, if *R*_*seal*_ is comparable to *R*_*patch*_, electrical shunt across the seal causes a depolarization shift of *E*_*m*_*rest*_ in CA. Closer agreement than here between *E*_*m*_*rest*_ in CA and WC in megakariocytes (Mason et al., 2005) was probably due to higher *R*_*seal*_ contact between the patch pipette and cell membrane, which is easier to achieve when patching isolated cells than neurons in brain slices. Resting *E*_*m*_ in CA values (up to −120 mV) that were more negative than *E*_*m*_*rest*_ in WC, are hardly explained by the error introduced by leakage via *R*_*seal*_, however. In addition, the CA/CC recordings were characterized by high levels of background noise. It is conceivable that these phenomena are caused by the high sensitivity of *E*_*m*_ in CA/CC to active currents across the patch membrane. It should be noted that the transmembrane potential in the attached membrane patch in CA/CC mode is nearly zero (because *E*_*m*_*ca*_ on the outer side of the membrane is close to the value of *E*_*m*_ inside the cell). Therefore, this highly depolarized membrane patch can be expected to exhibit activation of potassium channels and inactivation of sodium and calcium channels, as well as an increase in the driving force of outwardly directed currents through potassium and chloride channels, and a decrease in the driving force of inwardly directed currents through sodium and calcium channels. Thus, potassium conductance probably dominates in the membrane patch under these conditions, resulting in outwardly directed currents. These outward-directed currents, however, are seen as inward currents by the patch pipette attached to the cell, and thus should cause depolarizing signals in CA/CC contributing to depolarized *E*_*m*_*rest*_ values in CA/CC (in addition to the depolarization caused by leaky conductance via *R*_*seal*_). Note that even small currents can cause significant voltage changes in the patch. For example, opening a 100 pS potassium channel at a driving force for potassium of +90 mV generates a current of 9 pA, which induces a +9 mV depolarization of a 1GOhm patch. This active potassium conductance could contribute to depolarize *E*_*m*_*ca*_ relatively *E*_*m*_*wc*_, in addition to leakage through *R*_*seal*_. Less frequent but existing cases where *E*_*m*_*ca*_ values were more negative than *E*_*m*_*wc*_ are more difficult to explain. This could involve the presence of non-inactivating sodium or calcium channels in the patch mediating hyperpolarizing *E*_*m*_*ca*_ currents across the patch membrane, but this remains speculative. Sporadic opening and closing of ion channels also likely contributes to the high baseline fluctuations in the CA/CC recordings.

### Dynamic changes in membrane potential

Dynamic changes in *E*_*m*_ evoked by current injection via the WC-electrode, elevation of extracellular potassium, and during synaptic activity and APs were also detected during CA/CC recordings, but their amplitude in CA/CC was smaller than in WC. Overall, faster events were strongly attenuated. Constant or very slow shifts in *E*_*m*_ occurring at <1 Hz had a transfer coefficient on the order of 0.8–0.9. Transfer coefficients for faster events reduced to nearly 0.5 for synaptic potentials and 0.1 for APs. From a practical point of view, detection of PSPs was more difficult in CA/CC recordings because of the low signal-to-noise ratio due to the reduced amplitude of PSPs and the larger baseline fluctuations. Besides reduction in the amplitude, fast signals were also slowed down in their kinetics. These modifications in *E*_*m*_ signals were in keeping with the frequency-dependent changes in the transfer coefficient of the responses evoked by injection of sinusoidal current at different frequency. These changes in the fast signals corresponded to frequency-dependent changes in the transfer coefficient in the responses evoked by sinusoidal current injection at different frequencies. The frequency dependence of the transfer coefficient could be fitted by a model (Figures 5, 6). In general, these observations are in agreement with the results of previous studies (Fenwick et al., 1982; Tyzio et al., 2003; Hayar et al., 2004; Mason et al., 2005; Perkins, 2006), but provide more quantitative descriptions of the accuracy of CA/CC recordings for the assessment of biologically relevant *E*_*m*_ changes during synaptic events and action potentials in neurons.

CA/CC recordings also revealed dynamic changes in *E*_*m*_ during network driven GDPs in CA3 neurons from neonatal rat hippocampal slices and during SWRs in CA1 cells from adult mouse hippocampal slices. All neonatal CA3 hippocampal neurons depolarized during GDPs, and the amplitude of GDPs (16 mV, on average) was comparable to that obtained during gramicidin perforated patch recordings (also 16 mV, on average). This is slightly different from a prediction of a slight attenuation of GDPs in CA/CC recordings, because CA/WC amplitude transfer coefficient at GDPs waveform at their dominant frequency (3 Hz) is ~ 0.6 (Figure 6A). In addition, CA3 neurons fired bursts of APs during GDPs, and APs rate was maximal at the peak of field GDP. The temporal distribution of APs during GDPs in CA/CC recordings corresponded to the firing of multiple units and was also similar to the AP firing of CA3 neurons in gramicidin perforated patch recordings and during CA voltage clamp recordings as reported previously (Leinekugel et al., 1997; Khalilov et al., 2015). This differs from intracellular recordings with high chloride solution, where the AP firing occurs mainly during the rising and falling phases of GDPs, and depolarization block of APs at the at the peak of GDPs probably due to chloride overload of the cells. In contrast to GDPs, behavior of CA1 pyramidal cells during SWRs in adult hippocampal slices was more heterogeneous. Whereas, half of CA1 neurons depolarized during SWRs, another half of CA1 pyramidal neurons hyperpolarized during SWRs. Of the neurons depolarized during SWRs, 2 out of 5 (40%) fired APs. These findings are in agreement with intracellular recordings, which revealed about the same distribution of CA1 pyramidal cells behaviors during SWRs *in vitro* (Maier et al., 2003). In the latter study, the amplitude of SWRs was approximately 3 to 5 mV (see Figure 12 from Maier et al., 2003). Because the CA/WC amplitude transfer coefficient at the dominant frequency of SWRs waveform (7 Hz) is close to 0.5 (Figure 6A), SWRs amplitude of ~1.5 mV obtained in the present study using CA/CC recordings should be about half of “true” SWR amplitude, which is thus in agreement with the results of previous intracellular recordings. Together, these findings suggest that CA/CC recordings are appropriate for non-invasive assessment of *E*_*m*_ changes during network-driven activities, but stronger attenuation of faster signals should be considered.

### Polarity of GABAergic postsynaptic potentials

The undisturbed intracellular milieu is the main advantage of CA over WC recordings. In addition, despite artifacts affecting the measurement of *E*_*m*_*rest*_, CA recordings affect the actual *E*_*m*_*rest*_ very little. These are the two major requirements for assessing the polarity of postsynaptic responses, whose reversal potential is close to *E*_*m*_*rest*_, including those of GABA. Previously, CA/CC recordings have been used to demonstrate the predominantly positive polarity of puff-applied GABA responses in the L2/3 neocortical neurons of neonatal (P3–4) mice *in vivo*, and isoelectric responses to GABA in the presence of the membrane-permeable carbonic anhydrase inhibitor 6-ethoxy-2-benzothiazolesulfonamide in juvenile mice (P25–27) (Kirmse et al., 2015). Biphasic *E*_*m*_ shifts (hyperpolarizing to depolarizing) during GABA(A) receptor mediated giant postsynaptic potentials induced by 4-AP have also been described using CA/CC recordings from CA1 pyramidal cells in adult rat hippocampal slices (Perkins, 2006). Our findings provide direct evidence for the accuracy of CA/CC recordings in assessing the polarity of GABAergic responses. Indeed, we found that during dual WC and CA recordings, GABAergic postsynaptic responses display concomitant *E*_*m*_ - dependent change in polarity and show a dependence on [Cl^−^]_i_. Furthermore, consistent with previous observations, GABAergic postsynaptic responses were predominantly depolarizing during CA/CC recordings of intact neonatal CA3 pyramidal cells. Of note, the latter findings of depolarizing action of GABA at P6 are more consistent with *DF*_*GABA*_ and *E*_*GABA*_ non-invasive estimates using cell-attached GABA and NMDA channel recordings, and gramicidin perforated patch recordings, rather than using intracellular recordings with sharp electrodes. Indeed, in P6 CA3 pyramidal neurons, GABA exerts hyperpolarizing action and GDPs are associated with large hyperpolarizing potentials in intracellular recordings (Ben-Ari et al., 1989). These apparent contradictions are likely due to neuronal depolarization caused by the introduction of a leak conductance (~500 MOhm) using sharp electrodes, and modifications of [Cl^−^]_i_ due to dialysis as discussed in detail elsewhere (Khazipov et al., 2015). Thus, affecting neither *E*_*m*_ nor [Cl^−^]_i_, CA/CC recordings appear to be reliable, simple and rapid for assessing the polarity of GABAergic responses, which could be useful in physiological and pathological conditions when GABA polarity is challenged (Miles et al., 2012; Kaila et al., 2014; Khazipov et al., 2015; Akita and Fukuda, 2020; Cherubini et al., 2021). Although not investigated in this paper, the CA/CC approach may prove particularly useful for non-invasive recordings of membrane potential and synaptic events in dendrites and axons as it allows the use of electrodes with a smaller tip than for conventional WC recordings from the neuronal body. Limitations of this approach are the inability to estimate *E*_*GABA*_ due to a lack of control over *E*_*m*_, and relatively low signal-to-noise ratio.

## Data availability statement

The original contributions presented in the study are included in the article/supplementary material, further inquiries can be directed to the corresponding author/s.

## Ethics statement

The animal study was reviewed and approved by Local Ethical Committee of Kazan Federal University (No24/22.09.2020) French National Institute of Health and Medical Research (APAFIS #16992-2020070612319346 v2).

## Author contributions

RK and AN conceived the project and wrote the paper. AV, FV-R, and AR performed the experiments. AN, AV, AE, and FV-R analyzed the data. All authors contributed to the article and approved the submitted version.

## Funding

This work was supported by Russian Science Foundation # 20-75-00055 (experiments and analysis), subsidy allocated to Kazan Federal University for the state assignment # 0671-2020-0059 in the sphere of scientific activities (development of Eview software) and performed in the framework of the Kazan Federal University Strategic Academic Leadership Program (PRIORITY-2030).

## Conflict of interest

The authors declare that the research was conducted in the absence of any commercial or financial relationships that could be construed as a potential conflict of interest.

## Publisher's note

All claims expressed in this article are solely those of the authors and do not necessarily represent those of their affiliated organizations, or those of the publisher, the editors and the reviewers. Any product that may be evaluated in this article, or claim that may be made by its manufacturer, is not guaranteed or endorsed by the publisher.
